# (*S*)-1-Methoxy­carbonyl-2-(4-nitro­phen­yl)ethanaminium chloride

**DOI:** 10.1107/S1600536808020874

**Published:** 2008-07-12

**Authors:** Xiao-Chun Wen

**Affiliations:** a Ordered Matter Science Research Center, College of Chemistry and Chemical Engineering, Southeast University, Nanjing 210096, People’s Republic of China

## Abstract

The title compound, C_10_H_13_N_2_O_4_^+^·Cl^−^, comprises a Cl^−^ anion and a protonated aminium cation. The crystal packing is stabilized by cation–anion N—H⋯Cl hydrogen bonds and N—H⋯O hydrogen bonds, building an infinite two-dimensional network parallel to the (001) plane. The *S* absolute configuration at the chiral center was deduced from the synthetic pathway and confirmed by the X-ray analysis.

## Related literature

For details of α-amino acid derivatives as precursors for the synthesis of novel biologically active compounds, see: Lucchese *et al.* (2007 ▶); Arki *et al.* (2004 ▶); Hauck *et al.* (2006 ▶); Dai *et al.* (2008 ▶); Azim *et al.* (2006 ▶).
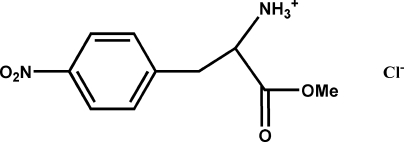


## Experimental

### 

#### Crystal data

C_10_H_13_N_2_O_4_^+^·Cl^−^
*M* *_r_* = 260.67
Monoclinic, 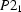

*a* = 4.825 (3) Å
*b* = 8.426 (3) Å
*c* = 15.111 (9) Å
β = 95.64 (4)°
*V* = 611.4 (6) Å^3^
*Z* = 2
Mo *K*α radiation
μ = 0.32 mm^−1^
*T* = 298 (2) K
0.25 × 0.18 × 0.17 mm

#### Data collection

Rigaku Mercury2 diffractometer
Absorption correction: multi-scan (*CrystalClear*; Rigaku, 2005 ▶) *T*_min_ = 0.931, *T*_max_ = 0.942
6215 measured reflections
2751 independent reflections
2077 reflections with *I* > 2σ(*I*)
*R*_int_ = 0.038

#### Refinement

*R*[*F* ^2^ > 2σ(*F* ^2^)] = 0.048
*wR*(*F* ^2^) = 0.112
*S* = 1.03
2751 reflections
154 parameters
1 restraint
H-atom parameters constrained
Δρ_max_ = 0.30 e Å^−3^
Δρ_min_ = −0.17 e Å^−3^
Absolute structure: Flack (1983 ▶), 1259 Friedel pairs
Flack parameter: −0.03 (9)

### 

Data collection: *CrystalClear* (Rigaku, 2005 ▶); cell refinement: *CrystalClear*; data reduction: *CrystalClear*; program(s) used to solve structure: *SHELXS97* (Sheldrick, 2008 ▶); program(s) used to refine structure: *SHELXL97* (Sheldrick, 2008 ▶); molecular graphics: *SHELXTL* (Sheldrick, 2008 ▶) and *Mercury* (Macrae *et al.*, 2006 ▶); software used to prepare material for publication: *SHELXTL*.

## Supplementary Material

Crystal structure: contains datablocks I, global. DOI: 10.1107/S1600536808020874/dn2364sup1.cif

Structure factors: contains datablocks I. DOI: 10.1107/S1600536808020874/dn2364Isup2.hkl

Additional supplementary materials:  crystallographic information; 3D view; checkCIF report

## Figures and Tables

**Table 1:** Hydrogen-bond geometry (Å, °)

*D*—H⋯*A*	*D*—H	H⋯*A*	*D*⋯*A*	*D*—H⋯*A*
N2—H11*B*⋯O4^i^	0.89	2.31	2.929 (4)	127
N2—H11*B*⋯Cl1^i^	0.89	2.71	3.380 (3)	133
N2—H11*C*⋯Cl1^ii^	0.89	2.42	3.175 (3)	143
N2—H11*A*⋯Cl1	0.89	2.34	3.151 (3)	151

Symmetry codes: (i) 
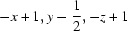
; (ii) 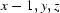.

**Fig. 1.**
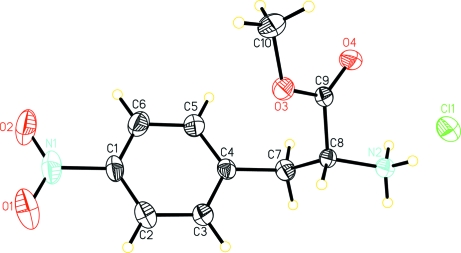
A view of the title compound with the atomic numbering scheme. Displacement ellipsoids were drawn at the 30% probability level. H atoms are represented as small spheres of arbitrary radii.

**Fig. 2.**
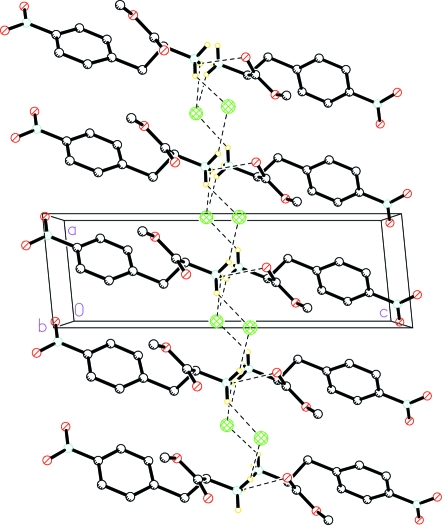
The crystal packing of the title compound viewed along the b axis and all hydrogen atoms not involved in hydrogen bonding (dashed lines) were omitted for clarity.

## supplementary crystallographic information

### Comment

α-Amino acid derivatives are important molecules due to their pharmacological properties. Recently, there has been an increased interest in the enantiomeric preparation of α-amino acid derivatives as precursors for the synthesis of novel biologically active compounds (Lucchese *et al.*, (2007); Arki *et al.*, (2004); Hauck *et al.*, (2006); Azim *et al.*, (2006); Dai *et al.*, (2008)). Here we report the crystal structure of the title compound.

The title compound is built up from a Cl^-^ anion and a protonated amino group cation (Fig. 1). The nitro group and the benzene ring are nearly planar, they are only twisted to each other by a torsion angles of C2-C1-N1-O1 (2.1 (7)° ) and C6-C1-N1-O2 (4.4 (7)° ), and the methyl 2-aminopropanoate substituent group is a zig-zag chain.

The crystal packing is stabilized by cation-anion N—H···Cl H-bonds and N—H···O H-bonds building an infinite two-dimensional network developping parallel to the (0 0 1) plane.(Table 1, Fig. 2).

The *S* absolute configuration at C8 is deduced from the synthetic pathway and confirmed by the X-ray analyses.

### Experimental

Under nitrogen protection, 2-amino-3-phenylpropanoic acid (30 mmol), nitric acid (50 mmol) and sulfuric acid (20 mmol) were added in a flask. The mixture was stirred at 110 °C for 3 h. The resulting solution was poured into ice water (100 mL), then filtered and washed with distilled water. The nitration amino acid was esterified with H_2_SO_4_ and CH_3_OH at 110 °C for 12 h, the crude product was obtained by evaporated the solution, and then recrystallized with distilled water by adding 1 ml HCl to yield colorless block-like crystals, suitable for X-ray analysis.

### Refinement

All H atoms attached to C atoms and N atoms were fixed geometrically and treated as riding with C—H = 0.96 Å (methyl), 0.97 Å (methylene), 0.98 Å (methine), 0.93 Å (aromatic) and N—H = 0.89 Å with U_iso_(H) = 1.2U_eq_(C except methyl) or U_iso_(H) = 1.5U_eq_(N and methyl C).

### Crystal data

**Table:**

C_10_H_13_N_2_O_4_^+^·Cl^–^	*F*_000_ = 272
*M**_r_* = 260.67	*D*_x_ = 1.416 Mg m^−^^3^
Monoclinic, *P*2_1_	Mo *K*α radiation λ = 0.71073 Å
Hall symbol: P 2yb	Cell parameters from 1445 reflections
*a* = 4.825 (3) Å	θ = 2.4–27.5º
*b* = 8.426 (3) Å	µ = 0.32 mm^−^^1^
*c* = 15.111 (9) Å	*T* = 298 (2) K
β = 95.64 (4)º	Block, colourless
*V* = 611.4 (6) Å^3^	0.25 × 0.18 × 0.17 mm
*Z* = 2	

### Data collection

**Table:**

Rigaku Mercury2 diffractometer	2751 independent reflections
Radiation source: fine-focus sealed tube	2077 reflections with *I* > 2σ(*I*)
Monochromator: graphite	*R*_int_ = 0.038
Detector resolution: 13.6612 pixels mm^-1^	θ_max_ = 27.4º
*T* = 298(2) K	θ_min_ = 2.7º
ω scans	*h* = −6→6
Absorption correction: multi-scan(CrystalClear; Rigaku, 2005)	*k* = −10→10
*T*_min_ = 0.931, *T*_max_ = 0.942	*l* = −19→19
6215 measured reflections	

### Refinement

**Table:**

Refinement on *F*^2^	Hydrogen site location: inferred from neighbouring sites
Least-squares matrix: full	H-atom parameters constrained
*R*[*F*^2^ > 2σ(*F*^2^)] = 0.048	*w* = 1/[σ^2^(*F*_o_^2^) + (0.0476*P*)^2^ + 0.0855*P*] where *P* = (*F*_o_^2^ + 2*F*_c_^2^)/3
*wR*(*F*^2^) = 0.112	(Δ/σ)_max_ < 0.001
*S* = 1.03	Δρ_max_ = 0.30 e Å^−^^3^
2751 reflections	Δρ_min_ = −0.17 e Å^−^^3^
154 parameters	Extinction correction: none
1 restraint	Absolute structure: Flack (1983), 1259 Friedel pairs
Primary atom site location: structure-invariant direct methods	Flack parameter: −0.03 (9)
Secondary atom site location: difference Fourier map	

### Special details

**Table:**

Geometry. All esds (except the esd in the dihedral angle between two l.s. planes) are estimated using the full covariance matrix. The cell esds are taken into account individually in the estimation of esds in distances, angles and torsion angles; correlations between esds in cell parameters are only used when they are defined by crystal symmetry. An approximate (isotropic) treatment of cell esds is used for estimating esds involving l.s. planes.
Refinement. Refinement of F^2^ against ALL reflections. The weighted R-factor wR and goodness of fit S are based on F^2^, conventional R-factors R are based on F, with F set to zero for negative F^2^. The threshold expression of F^2^ > 2sigma(F^2^) is used only for calculating R-factors(gt) etc. and is not relevant to the choice of reflections for refinement. R-factors based on F^2^ are statistically about twice as large as those based on F, and R- factors based on ALL data will be even larger.

### Fractional atomic coordinates and isotropic or equivalent isotropic displacement parameters (Å^2^)

**Table:**

	*x*	*y*	*z*	*U*_iso_*/*U*_eq_	
Cl1	0.98552 (15)	0.75535 (11)	0.54873 (5)	0.0499 (2)	
O3	0.8079 (4)	0.7690 (3)	0.30131 (12)	0.0483 (5)	
C9	0.6109 (5)	0.7495 (4)	0.35535 (16)	0.0362 (6)	
O4	0.4684 (5)	0.8525 (3)	0.38126 (14)	0.0498 (6)	
C8	0.5762 (6)	0.5766 (3)	0.37686 (18)	0.0353 (6)	
H8A	0.7584	0.5243	0.3803	0.042*	
N2	0.4629 (6)	0.5646 (3)	0.46458 (15)	0.0448 (6)	
H11A	0.5788	0.6120	0.5058	0.067*	
H11B	0.4447	0.4629	0.4787	0.067*	
H11C	0.2973	0.6119	0.4617	0.067*	
C4	0.4967 (6)	0.4821 (4)	0.21766 (19)	0.0400 (7)	
C7	0.3780 (7)	0.4943 (4)	0.3058 (2)	0.0439 (7)	
H7A	0.3369	0.3885	0.3262	0.053*	
H7B	0.2044	0.5530	0.2980	0.053*	
C1	0.7171 (8)	0.4557 (4)	0.0577 (2)	0.0493 (8)	
C2	0.8081 (8)	0.3560 (5)	0.1245 (2)	0.0576 (9)	
H2C	0.9440	0.2805	0.1165	0.069*	
C3	0.6962 (7)	0.3682 (4)	0.2046 (2)	0.0512 (8)	
H3A	0.7551	0.2991	0.2506	0.061*	
C5	0.4107 (7)	0.5812 (4)	0.1485 (2)	0.0530 (9)	
H5A	0.2761	0.6576	0.1561	0.064*	
C6	0.5198 (8)	0.5697 (5)	0.0674 (2)	0.0612 (10)	
H6A	0.4609	0.6375	0.0207	0.073*	
N1	0.8330 (9)	0.4439 (5)	−0.0288 (2)	0.0722 (10)	
O1	1.0166 (8)	0.3463 (5)	−0.0366 (2)	0.1027 (12)	
O2	0.7365 (9)	0.5296 (5)	−0.0890 (2)	0.1057 (12)	
C10	0.8487 (10)	0.9318 (4)	0.2717 (3)	0.0691 (11)	
H10A	0.9951	0.9340	0.2331	0.104*	
H10B	0.8984	0.9983	0.3224	0.104*	
H10C	0.6794	0.9702	0.2402	0.104*	

### Atomic displacement parameters (Å^2^)

**Table:**

	*U*^11^	*U*^22^	*U*^33^	*U*^12^	*U*^13^	*U*^23^
Cl1	0.0520 (4)	0.0476 (4)	0.0505 (4)	0.0037 (4)	0.0075 (3)	−0.0092 (4)
O3	0.0588 (12)	0.0420 (12)	0.0475 (11)	−0.0084 (12)	0.0217 (10)	0.0009 (12)
C9	0.0410 (14)	0.0359 (14)	0.0315 (12)	−0.0017 (16)	0.0027 (11)	−0.0029 (15)
O4	0.0582 (14)	0.0403 (12)	0.0521 (13)	0.0093 (11)	0.0123 (11)	0.0021 (11)
C8	0.0429 (16)	0.0342 (15)	0.0303 (14)	0.0008 (13)	0.0109 (12)	0.0018 (12)
N2	0.0599 (17)	0.0372 (14)	0.0390 (14)	−0.0001 (13)	0.0135 (12)	0.0005 (12)
C4	0.0466 (17)	0.0353 (15)	0.0389 (16)	−0.0111 (13)	0.0074 (14)	−0.0071 (13)
C7	0.0428 (17)	0.0444 (18)	0.0460 (17)	−0.0076 (14)	0.0114 (14)	−0.0031 (15)
C1	0.058 (2)	0.056 (2)	0.0364 (17)	−0.0125 (17)	0.0129 (15)	−0.0138 (15)
C2	0.060 (2)	0.062 (2)	0.052 (2)	0.0040 (19)	0.0099 (17)	−0.0160 (19)
C3	0.067 (2)	0.0434 (18)	0.0431 (18)	−0.0007 (17)	0.0073 (16)	−0.0024 (15)
C5	0.060 (2)	0.053 (2)	0.0465 (19)	0.0070 (17)	0.0083 (16)	0.0000 (17)
C6	0.078 (3)	0.064 (2)	0.0403 (19)	−0.006 (2)	0.0023 (18)	0.0038 (18)
N1	0.085 (3)	0.089 (3)	0.0451 (19)	−0.023 (2)	0.0180 (17)	−0.021 (2)
O1	0.094 (2)	0.141 (3)	0.078 (2)	0.008 (2)	0.0345 (19)	−0.035 (2)
O2	0.155 (3)	0.121 (3)	0.0465 (16)	−0.005 (3)	0.0353 (19)	0.0011 (19)
C10	0.090 (3)	0.052 (2)	0.068 (2)	−0.011 (2)	0.022 (2)	0.013 (2)

### Geometric parameters (Å, °)

**Table:**

O3—C9	1.323 (3)	C1—C2	1.353 (5)
O3—C10	1.462 (4)	C1—C6	1.371 (5)
C9—O4	1.196 (4)	C1—N1	1.475 (4)
C9—C8	1.506 (4)	C2—C3	1.377 (4)
C8—N2	1.486 (3)	C2—H2C	0.9300
C8—C7	1.532 (4)	C3—H3A	0.9300
C8—H8A	0.9800	C5—C6	1.384 (5)
N2—H11A	0.8900	C5—H5A	0.9300
N2—H11B	0.8900	C6—H6A	0.9300
N2—H11C	0.8900	N1—O2	1.217 (5)
C4—C5	1.370 (4)	N1—O1	1.223 (5)
C4—C3	1.387 (5)	C10—H10A	0.9600
C4—C7	1.505 (4)	C10—H10B	0.9600
C7—H7A	0.9700	C10—H10C	0.9600
C7—H7B	0.9700		
			
C9—O3—C10	115.6 (3)	C2—C1—C6	122.2 (3)
O4—C9—O3	125.7 (3)	C2—C1—N1	119.8 (4)
O4—C9—C8	123.5 (3)	C6—C1—N1	118.0 (4)
O3—C9—C8	110.8 (3)	C1—C2—C3	118.9 (3)
N2—C8—C9	108.4 (2)	C1—C2—H2C	120.6
N2—C8—C7	109.6 (2)	C3—C2—H2C	120.6
C9—C8—C7	111.2 (2)	C2—C3—C4	121.0 (3)
N2—C8—H8A	109.2	C2—C3—H3A	119.5
C9—C8—H8A	109.2	C4—C3—H3A	119.5
C7—C8—H8A	109.2	C4—C5—C6	121.4 (3)
C8—N2—H11A	109.5	C4—C5—H5A	119.3
C8—N2—H11B	109.5	C6—C5—H5A	119.3
H11A—N2—H11B	109.5	C1—C6—C5	118.2 (3)
C8—N2—H11C	109.5	C1—C6—H6A	120.9
H11A—N2—H11C	109.5	C5—C6—H6A	120.9
H11B—N2—H11C	109.5	O2—N1—O1	123.7 (4)
C5—C4—C3	118.4 (3)	O2—N1—C1	118.1 (4)
C5—C4—C7	121.4 (3)	O1—N1—C1	118.2 (4)
C3—C4—C7	120.2 (3)	O3—C10—H10A	109.5
C4—C7—C8	112.7 (3)	O3—C10—H10B	109.5
C4—C7—H7A	109.1	H10A—C10—H10B	109.5
C8—C7—H7A	109.1	O3—C10—H10C	109.5
C4—C7—H7B	109.1	H10A—C10—H10C	109.5
C8—C7—H7B	109.1	H10B—C10—H10C	109.5
H7A—C7—H7B	107.8		
			
C10—O3—C9—O4	−0.3 (4)	C1—C2—C3—C4	−1.1 (5)
C10—O3—C9—C8	176.6 (3)	C5—C4—C3—C2	0.9 (5)
O4—C9—C8—N2	−29.2 (4)	C7—C4—C3—C2	−179.7 (3)
O3—C9—C8—N2	153.9 (2)	C3—C4—C5—C6	−0.4 (5)
O4—C9—C8—C7	91.4 (3)	C7—C4—C5—C6	−179.9 (3)
O3—C9—C8—C7	−85.5 (3)	C2—C1—C6—C5	−0.4 (6)
C5—C4—C7—C8	−103.2 (4)	N1—C1—C6—C5	−180.0 (3)
C3—C4—C7—C8	77.3 (4)	C4—C5—C6—C1	0.2 (5)
N2—C8—C7—C4	−172.3 (2)	C2—C1—N1—O2	176.2 (4)
C9—C8—C7—C4	67.8 (3)	C6—C1—N1—O2	−4.2 (5)
C6—C1—C2—C3	0.8 (6)	C2—C1—N1—O1	−2.3 (5)
N1—C1—C2—C3	−179.6 (3)	C6—C1—N1—O1	177.3 (4)

### Hydrogen-bond geometry (Å, °)

**Table:**

*D*—H···*A*	*D*—H	H···*A*	*D*···*A*	*D*—H···*A*
N2—H11B···O4^i^	0.89	2.31	2.929 (4)	127
N2—H11B···Cl1^i^	0.89	2.71	3.380 (3)	133
N2—H11C···Cl1^ii^	0.89	2.42	3.175 (3)	143
N2—H11A···Cl1	0.89	2.34	3.151 (3)	151

Symmetry codes: (i) −*x*+1, *y*−1/2, −*z*+1; (ii) *x*−1, *y*, *z*.
